# Photoresponsive Type III Porous Liquids

**DOI:** 10.1002/chem.202202848

**Published:** 2022-12-01

**Authors:** Michael C. Brand, Nicola Rankin, Andrew I. Cooper, Rebecca L. Greenaway

**Affiliations:** ^1^ Department of Chemistry Materials Innovation Factory University of Liverpool 51 Oxford Street Liverpool L7 3NY UK; ^2^ Leverhulme Research Centre for Functional Materials Design Materials Innovation Factory and Department of Chemistry University of Liverpool Liverpool L7 3NY UK; ^3^ Department of Chemistry Molecular Sciences Research Hub Imperial College London 82 Wood Lane London W12 0BZ UK

**Keywords:** gas uptake, ionic liquids, metal–organic frameworks, photoresponsive, porous liquids

## Abstract

Porous materials are the subject of extensive research because of potential applications in areas such as gas adsorption and molecular separations. Until recently, most porous materials were solids, but there is now an emerging class of materials known as porous liquids. The incorporation of intrinsic porosity or cavities in a liquid can result in free‐flowing materials that are capable of gas uptakes that are significantly higher than conventional non‐porous liquids. A handful of porous liquids have also been investigated for gas separations. Until now, the release of gas from porous liquids has relied on molecular displacement (e.g., by adding small solvent molecules), pressure or temperature swings, or sonication. Here, we explore a new method of gas release which involves photoisomerisable porous liquids comprising a photoresponsive MOF dispersed in an ionic liquid. This results in the selective uptake of CO_2_ over CH_4_ and allows gas release to be controlled by using UV light.

## Graphical abstract

**Porous liquids** have typically focused on static components which require temperature and pressure swings, or sonication, to release gas from the system. Here, a new photoresponsive type III porous liquid is reported, formed by dispersing a known photoresponsive metal–organic framework in an ionic liquid, creating a porous liquid capable of releasing gas upon irradiation with UV light.

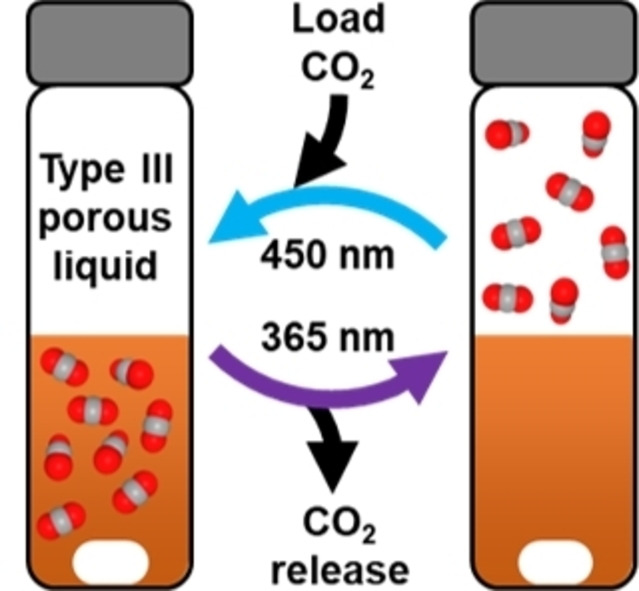

## Introduction

Porous liquids are a class of porous material that has recently caught the attention of researchers. First envisaged in 2007 by James and co‐workers,^[1]^ they later became a reality in 2015 where the first porous liquids were realised in the laboratory.[^2^, ^3^] These liquids differ from traditional solvents by having intrinsic micropores, allowing these materials to retain permanent porosity that leads to increased gas solubility and, in some cases, gas selectivity; that is, properties that are difficult to achieve with conventional non‐porous liquids.[^4^, ^5^, ^6^, ^7^] Porous liquids can be classified by the following categories: Type I porous liquids are discrete liquid hosts where the liquid component has shape‐persistent molecular porosity;[^8^, ^9^] Type II porous liquids are prepared by dissolving discrete porous molecules, such as porous organic cages (POCs), in a solvent that is excluded from the pores;[^10^, ^11^] Type III porous liquids are dispersions of porous solid particles, such as metal–organic frameworks (MOFs), in a fluid where the solvent is excluded from the pores;[^12^, ^13^] Type IV porous liquids are materials with extended connectivity in three dimensions that retains porosity in the liquid state.^14,15^ Since their conceptualisation, there have now been reports of all four types of porous liquids.[^9^, ^14^, ^16^, ^17^, ^18^]

While porous solids are applicable in a wide range of applications, including molecular separations and catalysis,[^19^, ^20^, ^21^] liquids have potential differentiable advantages because they can flow and might also promote more rapid heat transfer and dissipation.^[22]^ By contrast, the porosity per unit volume for porous liquids is thus far much lower than for porous solids. As such, porous liquids might allow for new, different processes, rather than simply replacing porous solids. There are already excellent examples of what conventional (non‐porous) liquid solvents can achieve in industrial processes such as ‘wet‐scrubbing’ for CO_2_ capture, which involves aqueous amine solutions to chemically absorb CO_2_.^[23]^ However, this typically requires large amounts of energy to regenerate the liquid.^[24]^ Porous liquids, on the other hand, might be adapted into existing infrastructure that uses conventional flow processes, while also lowering the energy penalty of solvent regeneration by exploiting physisorption rather than chemisorption.[^25^, ^26^] In addition, the incorporation of discrete molecules or frameworks into a liquid also adds the potential for shape and size specificity, allowing porous liquids to be tailored towards target separations and applications.[^27^, ^28^]

To date, porous liquids have been mostly studied for their ability to absorb gas, and only a handful of materials have been explored for gas selectivity. So far, little research has gone into understanding the gas release mechanisms of porous liquids. Several methods for controlled gas release from porous liquids have been reported, each providing its own challenges. As for porous solids, the most common gas release mechanisms are pressure and temperature swings.[^11^, ^29^, ^30^] This significantly limits the solvent choice in a porous liquid, restricting us to solvents that have a low or (ideally) near‐zero vapour pressure.[^11^, ^31^] There is also a significant parasitic energy penalty with pressure and temperature swings. Moreover, some materials might not be stable to temperature swings; for example, it was reported that some porous liquids are unable to withstand temperature displacement over multiple cycles.^[11]^ An alternative method to release gases is based on guest exchange by chemical displacement, where the addition of a small molecule with a more favourable binding in the cavity is used to displace the gaseous guest from the pores.[^11^, ^32^, ^33^] While effective in the laboratory, this method has very limited practical appeal because the displacing molecule is harder to remove than the gas. Lastly, sonication has been used to release gaseous guests from porous liquids.^[30]^ While this method is a non‐invasive alternative to chemical displacement, the relative parasitic energy costs are as yet unclear and more problematically, sonication may be undesirable for large scale processes (e.g., cavitation can damage metal containment vessels).^[34]^

**Figure 1:**
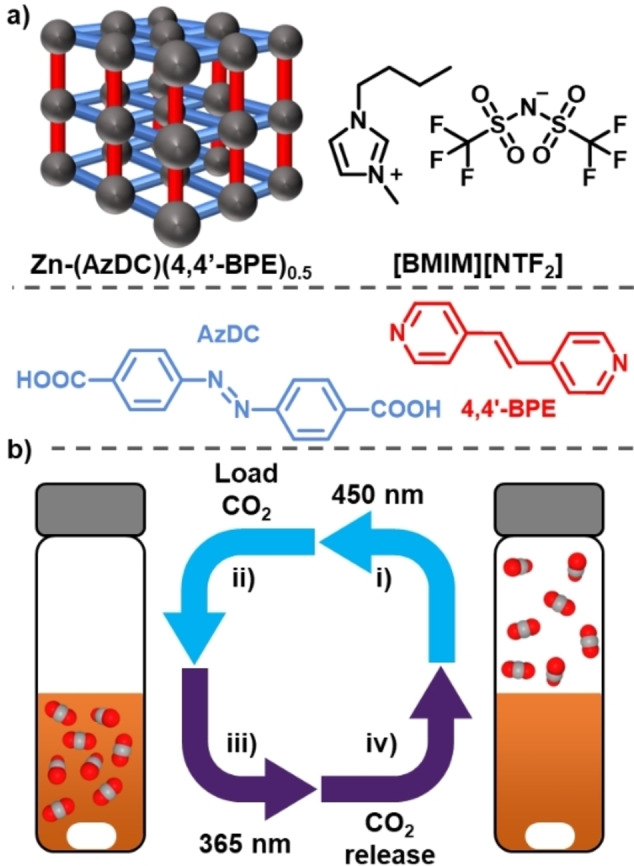
a) Illustration of the components used to form a photoresponsive Type III porous liquid from the MOF Zn(AzDC)(4,4‘‐BPE)_0.5_ and the ionic liquid [BMIM][NTf_2_]; b) Illustration of the uptake and release cycle for CO_2_ in the photoresponsive porous liquid.

Here, we investigate the formation of photoresponsive Type III porous liquids. We exploited well‐known photochromic molecules, azobenzene and 1,2‐bis(4‐pyridyl)ethylene, both of which have been incorporated into a zinc‐based MOF (Figure 1a) that is capable of efficient and reversible *cis* to *trans* photoisomerisation when exposed to UV or visible light (*trans*: *λ*_max_ ≈370 nm, *cis λ*_max_≈460 nm). This system was previously reported by Chen et al. and later studied by Lyndon et al. for its dynamic photoswitching in the solid state; specifically a change in CO_2_ uptake was reported upon photoswitching.[^35^, ^36^] By dispersing this photoresponsive MOF in an ionic liquid as the size‐excluded solvent, we have developed a new Type III porous liquid, creating what we believe to be the first proof‐of‐concept photoresponsive porous liquid that is capable of selective gas uptake and UV light‐triggered release (Figure 1b).

## Results and Discussion

First, we built a suitable photoreactor that would allow us to carry out sorption measurements while irradiating the sample (Figure S1). For this, we designed a reactor that could accommodate a gas sorption tube and light source, with a stirrer plate positioned underneath the porous liquid sample during the gas sorption experiment (Figure S2). This design was subsequently 3D printed using a resin printer. The first prototypes used an LED light source that did not produce enough power output and that also caused an increase in the internal temperature in the reactor. This was mitigated by using an external light source in tandem with fans mounted to either side of the reactor, with small passages to create a push‐pull airflow through the reactor. The temperature was monitored over the course of several experiments, and it remained in the range 28.5±0.5 °C, regardless of whether the light source (365 nm) was switched on (Figure S3). The CAD design and 3D print files are available at github.com**/**GreenawayLab/Sorption‐Photoreactor.

The photoresponsive MOF Zn(AzDC)(4,4‘‐BPE)_0.5_ was chosen based on previous reports that it exhibits a decrease in gas uptake when exposed to UV light: a difference of 64 % was found under dynamic conditions and 42 % under static conditions.^[36]^ In addition, this material was noted for having selective adsorption for carbon dioxide over methane. In our hands, the CO_2_ uptake of this MOF varied considerably between batches, ranging from 277‐766 μmol/g at 1 bar/301 K, even when the synthesis and activation was carried out on the same scale (Figure S6), though there was little variation in the measured particle size distributions of the three batches selected for further analysis (Figure S17, Table S4). It is therefore possible that the variation in CO_2_ uptake is due to the formation of differing quantities of triply, double, and non‐penetrating frameworks, where differences can be seen in the PXRD patterns which deviate from the reported triply‐interpenetrated framework (Figure S6).^[35]^ For the batch with the largest CO_2_ uptake, a reduction in CO_2_ uptake of 31 % was achieved under static irradiation conditions at 365 nm with 100 % exposure in the 3D printed reactor (CO_2_ uptake, 1 bar: 766 μmol/g, ambient; 526 μmol/g under UV irradiation).

Next, a suitable solvent was selected to produce a Type III porous liquid. To date, a wide variety of liquids have been used, including organic solvents, oils, and ionic liquids.[^37^, ^38^] For this study, we screened a small number of liquids that have previously been reported as being size‐excluded for POCs and MOFs, including silicone oil AR 20, and the ionic liquids 1‐butylpyridinium bis(trifluoromethanesulfonyl)imide ([BPy][NTf_2_]), 1‐ethyl‐3‐(hydroxymethyl) pyridinium ethyl sulfate ([EtHPy][EtS]), and 1‐butyl‐3‐methylimidazolium bis(trifluoromethylsulfonyl)imide ([BMIM][NTf_2_]). Ionic liquids are good candidates for porous liquids due to their low vapour pressures, compatibility with organic and inorganic materials, and their ability to be tailored to suit different applications. To screen these solvents for size‐exclusion from the MOF, 12.5 wt. % dispersions were prepared, and the CO_2_ uptake was measured under ambient conditions (i.e., with no exposure to UV or blue light) while stirring at 300 rpm (Figure S5). While several of these combinations showed enhanced CO_2_ uptake compared to the neat liquids, which indicated the successful formation of Type III porous liquids, silicone oil AR 20 and [BMIM][NTf_2_] demonstrated the highest CO_2_ uptakes when compared to the neat liquids. [BMIM][NTf_2_] was selected due to the larger increase in CO_2_ uptake on going from the neat liquid to the 12.5 wt. % dispersion. The porous liquid also demonstrated excellent CO_2_/CH_4_ selectivity, whereas the silicone oil had a CH_4_ uptake that was four times greater than the neat ionic liquid [BMIM][NTf_2_]. In addition, for the specific sample of MOF and batch of ionic liquid used in this screen, the CO_2_ uptake in the 12.5 wt. % dispersion corresponded to 74 % of the theoretical total uptake, based on the calculated CO_2_ uptake from the proportion of the MOF (749 μmol/g) combined with the uptake of the neat liquid (83 μmol/g_L_); that is, the porous liquid expresses 74 % of the total uptake that would be expected assuming a linearly additive system upon mixing.

**Figure 2:**
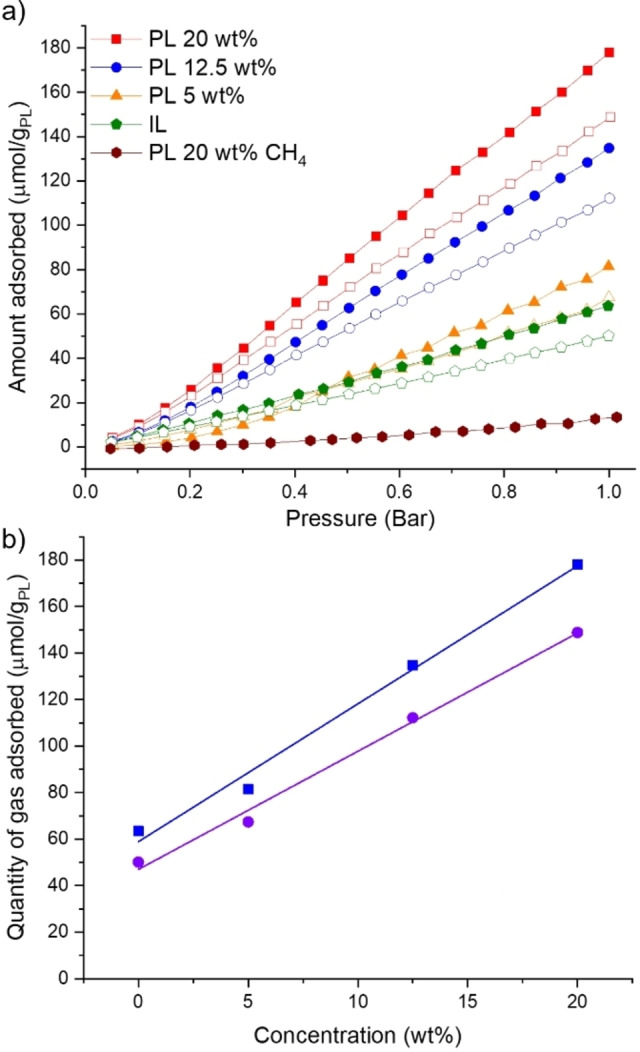
a) CO_2_ adsorption isotherms for Zn(AzDC)(4,4‘‐BPE)_0.5_ [BMIM][NTf_2_] porous liquid and neat [BMIM][NTf_2_] ionic liquid under ambient conditions (filled) and UV light (emtpy); b) Measured gas uptake across a range of concentrations of porous liquid, under ambient conditions (blue squares) and constant irradiation (purple circles), with the corresponding linear fit lines.

To investigate the photoresponsive nature of the porous liquid formed with ionic liquid [BMIM][NTf_2_], we prepared a series of 5, 12.5 and 20 wt. % samples (based on the MOF loading) directly in gas sorption tubes and measured the CO_2_ uptake. First, the batch of MOF with the highest CO_2_ uptake (766 μmol/g) was investigated. We observed a proportionate increase in CO_2_ uptake upon increasing the wt. % loading of the MOF in the porous liquid (Figure 2; Table S1). The CO_2_ uptake measurements were then repeated for each porous liquid sample while exposing them to UV light (365 nm) for the full duration of the sorption experiment. All samples demonstrated a consistent decrease in CO_2_ uptake at 1 bar, with decreases of 17 %, 16 %, and 16 % observed for samples with MOF loadings of 5, 12.5 and 20 wt. %, respectively (Figure 2b).

A decrease in CO_2_ solubility was also observed in the neat ionic liquid [BMIM][NTf_2_] upon UV irradiation (63 μmol/g_L_, ambient; 50 μmol/g_L_, UV irradiation). Since the temperature in the photoreactor remained constant during the sorption measurements, it is unlikely that this was the cause of decrease in CO_2_ solubility. To our knowledge, there are few studies investigating the effects of UV irradiation on ionic liquids. However, a report by Rao et al.^[39]^ notes changes in the UV‐Vis and fluorescence spectra on a similar ionic liquid, [BMIM][BF_4_], but concludes that there is very little change in the bulk of the material. For [BMIM][NTf_2_], changes are observed in the UV‐Vis spectra on irradiation (Figure S20), which may be responsible for the observed changes in gas solubility, but no structural changes were observed in the ^1^H NMR spectra (Figure S21). In addition, the difference in CO_2_ uptake upon irradiation in the ionic liquid (13 μmol/g_L_) was lower than for the porous liquids at higher MOF loadings (12.5 wt. %, 23 μmol/g_PL_, 20 wt. %, 29 μmol/g_PL_), confirming an additional contribution from the incorporated MOF.

Given the observed difference in CO_2_ uptake across the different batches of MOF (Figure S6), we were interested to see whether there was a direct correlation with the resulting uptake in the subsequent porous liquid. Therefore, 5, 12.5, and 20 wt. % porous liquid samples incorporating two further batches of MOF with CO_2_ uptakes of 457 μmol/g (Figure S11–S12, Table S2) and 277 μmol/g (Figure S13–S14, Table S3) were investigated. As might be expected for a Type III porous liquid where the solid pore carrier is simply dispersed, there was a linear trend between the measured uptake in the solid MOF and the resulting uptake in the porous liquid under ambient conditions (Figure S16). Similarly, the reduction in CO_2_ uptakes was consistent under UV irradiation across the different samples (Figure S11, S14). The porous liquids investigated (using the three different batches of MOF with uptakes of 277, 457 and 766 μmol/g), both under ambient conditions and under UV irradiation, demonstrated at least 82 % of the theoretical calculated total uptakes. The MOF also retained its bulk crystallinity after being dispersed in the ionic liquid and after gas uptake measurements under both ambient conditions and under UV irradiation (Figure S9, S12, S15). In addition, on sonicating the MOF in [BMIM][NTf_2_] a visibly stable dispersion was formed, which showed good stability with no obvious creaming or sedimentation occurring on standing over several months (Figure S18).

**Figure 3:**
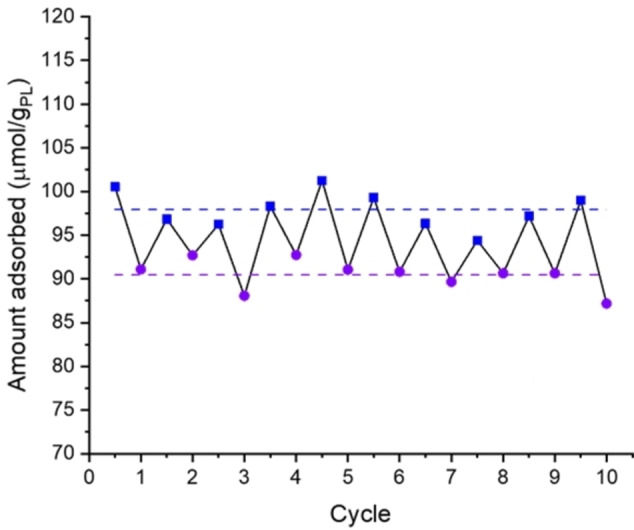
Recycling study of the 12.5 wt. % porous liquid for CO_2_ uptake with UV light on (365 nm, purple circles) and light off (exposed to 450 nm for 10 minutes, blue squares).

We next investigated the cyclability and reproducibility of CO_2_ uptake in the photoresponsive porous liquid on switching between UV (365 nm) and blue (450 nm) light (Figure 1b). A 12.5 wt. % porous liquid sample was prepared and cycled through 10 experiments where it was continually exposed to UV light for the ‘on’ experiments, before being exposed to blue (450 nm) light for 10 minutes and then starting the ‘off’ experiment (Figure 3). Overall, the average CO_2_ uptake was 97.9±2.0 μmol/g_PL_ after the porous liquid was exposed to blue light, compared to 90.4±1.6 μmol/g_PL_ when the porous liquid was exposed to UV light ‐ this is a decrease of 7.5 μmol/g_PL_, equating to an 8 % reduction of CO_2_ in the porous liquid when exposed to UV light. While this was lower than the previously observed 16 %, we believe this is due to partial saturation since the sample was not left to degas for 24 h, unlike the initial experiments, but rather was only degassed for 60 minutes between measurements. Hence the sample is likely still partially saturated with gas leading to a lower CO_2_ capacity and smaller difference when the sample is exposed to UV light.

We also studied the gas selectivity of this porous liquid. First, the CH_4_ uptake was investigated in the neat ionic liquid and in the 20 wt. % porous liquids formed using the three different batches of MOF. Little to no CH_4_ uptake was observed, and all the porous liquid uptakes were well below that of the theoretical gas uptakes (Table S1, S2 and S3). For the MOF with the highest uptake in the solid state, there was a very small increase in CH_4_ uptake from 4.0 μmol/g_PL_ in the neat ionic liquid to 13.4 μmol/g_PL_ in the porous liquid; this demonstrates a 13‐fold increase of CO_2_ over CH_4_ for the 20 wt. % porous liquid, which we believe is the highest CO_2_/CH_4_ selectivity of any reported porous liquid to date. We ascribe the lack of CH_4_ uptake in the porous liquid to the very low solubility of methane in the ionic liquid carrier solvent and the preferential CO_2_ uptake observed in the photoresponsive MOF in the solid‐state.

**Figure 4:**
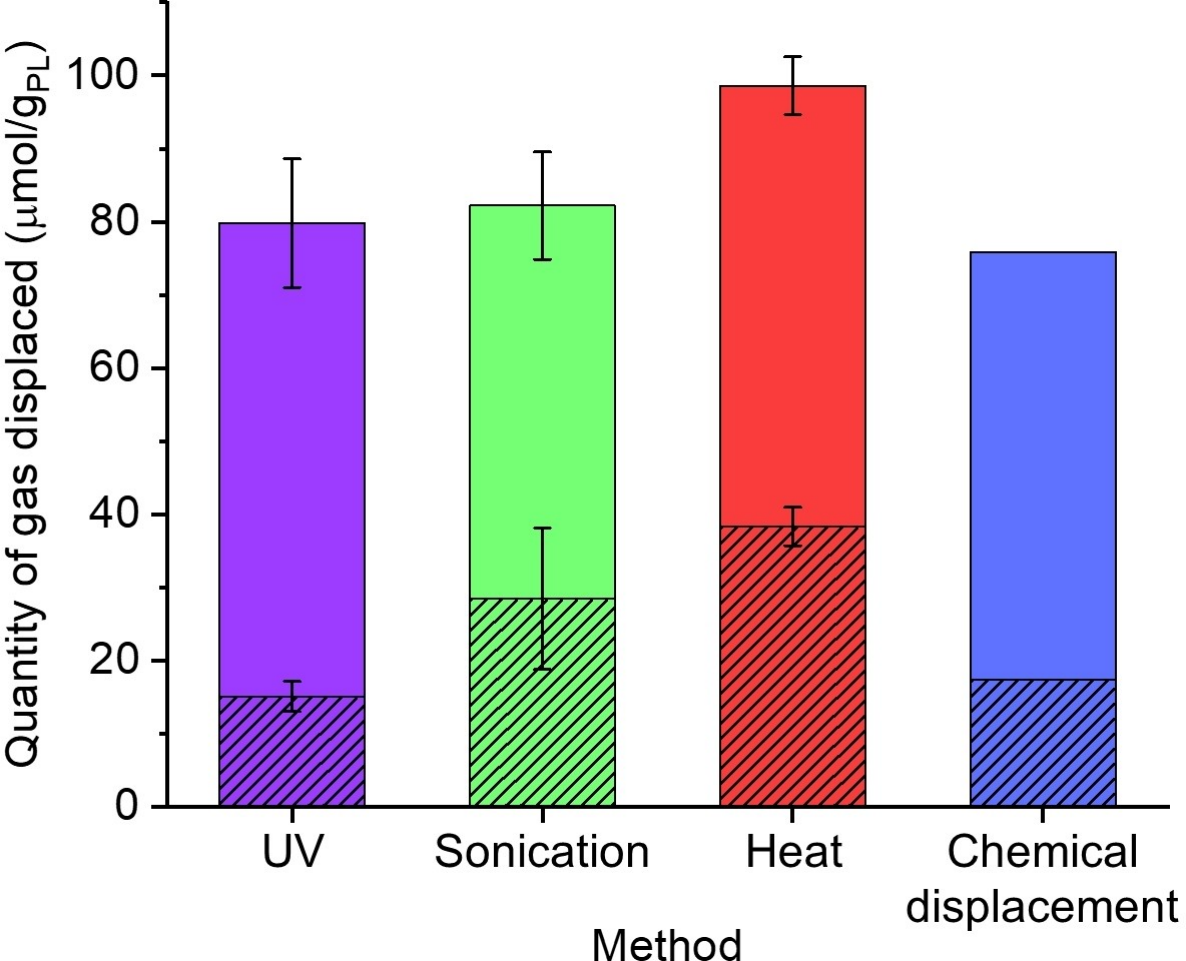
Gas displacement experiments for CO_2_ using different mechanisms of release, including UV, sonication, heating (80 °C), and chemical displacement (0.4 mL CHCl_3_), in the 12.5 wt. % porous liquid (solid) and ionic liquid (dashed lines).

Finally, different methods of controlled gas release were investigated using CO_2_‐loaded porous liquid samples, as measured by collecting and measuring the amount of gas released (Figure 4; Figure S19, Tables S6–S7). This included irradiation at 365 nm while stirring, sonication in a room temperature water bath, heating and stirring at 80 °C, and chemical displacement by the addition of chloroform. Increasing the temperature of the sample to 80 °C resulted in the highest quantity of CO_2_ being released, displacing 98.6±3.9 μmol/g_PL_. Both sonication and UV irradiation performed equally well, displacing 82.2±7.3 μmol/g_PL_ and 79.8±8.7 μmol/g_PL_, respectively. Chemical displacement performed the least well of the methods tested, displacing 75.9 μmol/g_PL_. In all cases, the amount of gas released by the porous liquid was significantly higher than that of the neat ionic liquid and irradiation showed the largest difference, with a 426 % increase. The other release methods ranged from a 115 % to 341 % increase over the neat ionic liquid. This correlates with an 81 % difference in the quantity released between the porous liquid and the ionic liquid on irradiation, compared to 65 % for sonication, 61 % for thermal release, and 77 % for chemical displacement (Table S8). This shows that photochemical release is almost as effective as traditional methods, such as temperature swing, in terms of the total amount of gas released. However, when the difference in gas release is compared, UV‐irradiation is the most effective in terms of the working capacity, with thermal release the least effective due to it being a non‐specific trigger that is commonly used for liquid sorbents.

## Conclusion

In conclusion, a photoresponsive Type III porous liquid was formed by dispersing a MOF containing photoswitchable groups in a size‐excluded ionic liquid. Flowable dispersions up to 20 wt. % could be formed, and UV light was successfully used as a gas release mechanism. These porous liquids demonstrate enhanced CO_2_ uptake over the neat ionic liquid and exhibit excellent CO_2_/CH_4_ selectivity. While the CO_2_ uptake was found to vary between different batches of the synthesised MOF, a linear relationship was found between the uptake in the solid and the resulting uptake in the porous liquids. Taken together, these results suggest alternative paradigms for selective gas capture and release using material compositions that can flow.

## Experimental Section

Full experimental details can be found in the Supporting Information.

**Materials**: 4‐Nitrobenzoic acid, [BMIM][NTf_2_] and zinc nitrate were purchased from Sigma‐Aldrich, t*rans*‐1,2‐bis(4‐pyridyl)ethylene was purchased from Fluorochem, and all chemicals were used as received. Other solvents were reagent or HPLC grade purchased from Fisher Scientific.

**Synthesis of 4,4′‐(diazene‐1,2‐diyl)dibenzoic acid**: 4,4′‐(Diazene‐1,2‐diyl)dibenzoic acid was synthesised following the procedure reported by Lyndon et al.^[36]^ 4‐Nitrobenzoic acid (15 g, 89.8 mmol) was added to a solution of sodium hydroxide (51 g, 1.28 mol, in 225 mL water), and the solution was gently heated until the solid dissolved. Glucose (100 g, 55.5 mmol) was dissolved in 150 mL water and gently heated until the solid dissolved. The warm glucose solution was then added slowly and portion wise to the solution at 50 °C, where the solution initially formed a yellow precipitate, before turning brown upon further addition of glucose. The mixture was left to stir overnight at room temperature. Methanol was added to the solution until a light brown precipitate formed. The precipitate was collected by filtration, dissolved in water, and acidified with acetic acid (∼20 mL), and then filtered once more yielding a light pink precipitate. The product was washed with an excess of water and then dried under vacuum for several hours to yield the final product (6.5 g, 24.0 mmol, 53 %).

**Synthesis of MOF Zn(AzDC)(4,4’‐BPE)_0.5_**: The material was synthesized following the procedure reported by Chen et al. at double the scale, and repeated to form six different batches of MOF.^[35]^ Zn(NO_3_)_2_ ⋅ 6H_2_O (0.56 g, 1.88 mmol), 4,4′‐(diazene‐1,2‐diyl)dibenzoic acid (0.51 g, 1.88 mmol), and *trans*‐1,2‐bis(4‐pyridyl)ethylene (0.17 g, 0.94 mmol) were suspended in DMF (200 mL) and heated in a sealed 500 mL Duran Bottle at 100 °C for 24 h. The red crystals were then collected by filtration and washed with DMF, hexane, and then dried in air, yielding the final product as a red solid (0.65–0.79 g, 1.05–1.28 mmol, 56–68 % based on C_27.5_H_31.5_N_5.5_O_7_Zn). Prior to sorption measurements samples were left to stir in MeOH for 1 h, before the solid was collected by filtration and heated to 150 °C under dynamic vacuum for at least 24 h.

**Gas sorption measurements of the porous liquid**: The gas uptake of solid and liquid samples was measured on a Quantachrome Nova 4200e. Porous liquid samples were prepared in BET tubes by weighing solid MOF and adding the corresponding amount of liquid to prepare 5, 12.5 or 20 wt. % porous liquid samples. The samples were sonicated for 10 minutes and then degassed under dynamic vacuum while stirring overnight before backfilling with helium. After degassing, samples were weighed and placed on the analysis port. Sorption measurements were performed at ambient temperature (within a custom‐made 3D printed photoreactor box, github.com**/**GreenawayLab/Sorption‐Photoreactor) while being stirred at 300 RPM. Samples that required UV irradiation were also measured inside of the photoreactor box and irradiated with 365 nm light from an external light source with a light probe inside of the box.

## Conflict of interest

The authors declare no conflict of interest.

1

## Supporting information

As a service to our authors and readers, this journal provides supporting information supplied by the authors. Such materials are peer reviewed and may be re‐organized for online delivery, but are not copy‐edited or typeset. Technical support issues arising from supporting information (other than missing files) should be addressed to the authors.

Supporting Information

## Data Availability

The data that support the findings of this study are available in the supplementary material of this article.
