# Supplementary material for: Photoresponsive Type III Porous Liquids
Source: Chemistry. 2022 Dec 1;29(4):e202202848. doi: 10.1002/chem.202202848 (PMC10108065; doi:10.1002/chem.202202848)
Supplement: Supplementary file 1 — Supporting Information [file CHEM-29-0-s001.pdf]

Chemistry–A European Journal

Supporting Information

Photoresponsive Type III Porous Liquids

Michael C. Brand, Nicola Rankin, Andrew I. Cooper, and Rebecca L. Greenaway*

1. Supplementary Information Contents

1. Supplementary Information Contents 1
2. General Synthetic and Analytical Methods 2
3. Synthesis and Characterisation 3
4. Light-Responsive Porous Liquid Gas Adsorption Experimental Setup 4
5. Porous Liquid Solvent Screen 7
6. Material Reproducibility and Gas Sorption Studies 8
7. Gas Evolution Experimental setup 18
8. UV-Vis and NMR Study of [BMIM][NTf_2] 21
9. References 23

The following abbreviations have been used throughout the supporting information:

MOF	Metal-Organic Framework
AzDC	4,4'-(diazene-1,2-diyl)dibenzoic acid
4,4’-BPE	*trans*-1,2-bis(4-pyridyl)ethylene
PL	Porous Liquid
IL	Ionic Liquid
[BPy][NTf_2]	1-butylpyridinium bis(trifluoromethanesulfonyl)imide
[EtHPy][EtS]	1-ethyl-3-(hydroxymethyl) pyridinium ethyl sulfate
[BMIM][NTf_2]	1-butyl-3-methylimidazolium bis(trifluoromethylsulfonyl)imide

2. General Synthetic and Analytical Methods

Materials: 4-Nitrobenzoic acid, [BMIM][NTf_2] and zinc nitrate were purchased from Sigma-Aldrich, t*rans*-1,2-bis(4-pyridyl)ethylene was purchased from Fluorochem, and all chemicals were used as received. Other solvents were reagent or HPLC grade purchased from Fisher Scientific.

NMR: ^{1}H and ^{13}C Nuclear magnetic resonance spectra were recorded using an internal deuterium lock for the residual protons in DMSO-d6 (δ 2.50 ppm and 39.52 ppm) at ambient probe temperature on a Bruker Avance 400 (400 MHz/101 MHz) instrument. ^{1}H NMR data are presented as follows: chemical shift, peak multiplicity (s = singlet, d = doublet, t = triplet, q = quartet, m = multiplet, br = broad, app = apparent), coupling constants (J / Hz), and integration. Chemical shifts are expressed in ppm on a δ scale relative to δ_{DMSO} (2.50 ppm and 39.52 ppm) and coupling constants, J, are given in Hz

PXRD: Laboratory powder X-ray diffraction data were collected in transmission mode on samples held on thin Mylar film in aluminium well plates on a Panalytical X'Pert PRO MPD equipped with a high-throughput screening (HTS) XYZ stage, X-ray focusing mirror and PIXcel detector, using Ni-filtered Cu Kα radiation. Data were measured over the range 4–40° in ~0.013° steps over 60 minutes.

Static Light Scattering (SLS): The particle size distributions were determined using SLS measurements and were performed on a Malvern Instruments Mastersizer 3000. Samples were measured in methanol with constant stirring to ensure scattering intensity was within range. Particle size ranges are expressed as Dx10, Dx50, and Dx90, taken from the cumulative distribution which corresponds to the 10th, 50th, and 90th percentile of distribution.

Viscosity measurements: The viscosity of liquid samples were carried out on a TA Instruments Ares-G2 rheometer fitted with a 25 mm 0.1 rad cone attachment. Viscosities were measured at 25 °C with a constant shear rate of 300 s^{-1} with an experiment duration of 300 seconds. Data points were taken every 3 seconds and the viscosity is reported as an average of all data points.

3. Synthesis and Characterisation

Synthesis of 4,4'-(diazene-1,2-diyl)dibenzoic acid

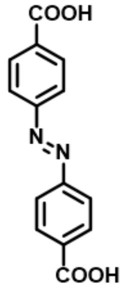

4,4'-(Diazene-1,2-diyl)dibenzoic acid was synthesised following the procedure reported by Lyndon *et al*.[1] 4-Nitrobenzoic acid (15 g, 89.8 mmol) was added to a solution of sodium hydroxide (51 g, 1.28 mol, in 225 mL water), and the solution was gently heated until the solid dissolved. Glucose (100 g, 55.5 mmol) was dissolved in 150 mL water and gently heated until the solid dissolved. The warm glucose solution was then added slowly and portion wise to the solution at 50 °C, where the solution initially formed a yellow precipitate, before turning brown upon further addition of glucose. The mixture was left to stir overnight at room temperature. Methanol was added to the solution until a light brown precipitate formed. The precipitate was collected by filtration, dissolved in water, and acidified with acetic acid (~20 mL), and then filtered once more yielding a light pink precipitate. The product was washed with an excess of water and then dried under vacuum for several hours to yield the final product (6.50 g, 24.0 mmol, 53%).

^{1}H NMR (400 MHz, DMSO-d6) δ_H 8.16 (d, J = 8.7 Hz, 4H), 8.01 (d, J = 8.7 Hz, 4H). No trace of acid peak. **^{13}C NMR** (101 MHz, DMSO-d6) δ_C 166.67, 154.17, 133.53, 130.71, 122.85. Data in accordance with literature.[1]

Synthesis of MOF Zn(AzDC)(4,4'-BPE)$_{0.5}$

Material was synthesised following the procedure reported by Chen *et al.* at double the scale, and repeated to form six different batches of MOF.[2] $Zn(NO_3)_2 \cdot 6H_2O$ (0.56 g, 1.88 mmol), 4,4'-(diazene-1,2-diyl)dibenzoic acid (0.51 g, 1.88 mmol), and *trans*-1,2-bis(4-pyridyl)ethylene (0.17 g, 0.94 mmol) were suspended in DMF (200 mL) and heated in a 500 mL Duran Bottle at 100 °C for 24 hours. The red crystals were then collected by filtration and washed with DMF, hexane, and then dried in air, yielding the final product as a red solid (0.65-0.79 g, 1.05-1.28 mmol, 56-68% based on $C_{27.5}H_{31.5}N_{5.5}O_7Zn$).

Prior to sorption measurements samples were left to stir in MeOH for 1 hour, before the solid was collected by filtration and heated to 150 °C under dynamic vacuum for at least 24 h.

4. Light-Responsive Porous Liquid Gas Adsorption Experimental Setup

Gas Sorption Analysis: Gas sorption measurements on porous liquids and ionic liquids were measured on a Quantachrome Nova 4200e. Solid and liquid samples were degassed separately in a vacuum oven overnight at 150 °C before being added to 9 mm BET tubes with a small glass stir bar (6 mm x 5 mm), and then sonicated for 10 minutes. Porous liquid samples were then degassed under dynamic vacuum while stirring overnight before backfilling with helium. Samples were then weighed after degassing and placed on the analysis port. Sorption measurements were then performed at room temperature while stirring at 300 RPM. Adsorption settings were as follows: 20 pressure points from 0.05 to 1.0 bar in 0.05 increments; pressure tolerance = 0.05 mmHg; equilibration time = 1800 seconds; equilibration timeout = 5400 seconds.[3]

To analyse samples while irradiating with UV-light, a custom-made 3D printed reactor was designed with mirror coated internal walls (acrylic mirror, 3mm thick) fitted with an external light source (Omnicure S1000, fitted with a 365 nm filter) and a light guide to maximise sample exposure during the experiment. As the sample needed to be stirred during the measurement to ensure equilibration and saturation, a stirrer plate was placed under the reactor box while operating. Two external 40 mm fans mounted either side in a push-pull configuration against the reactor walls with slits allow air to pass through the reactor. Temperature within the box was monitored with a thermocouple and points were taken at several times over the day over several experiments and was found to maintain a temperature between 28 and 29 °C which fluctuated less than 1 °C, regardless of whether the light source was on or not (**Supplementary Figure 3**). During the experiment, the UV light source was set to the maximum intensity and was continuous throughout. For ‘ambient’ conditions, the sample was irradiated with a blue (450 nm) LED for 15 minutes prior to starting the experiment.

3D Printers: A Form 3 resin printer from Formlabs was used to print the photoreactor and probe mount in white resin (GPWH04). Prints were washed for 15 minutes in an IPA bath and then cured for 30 minutes at 60 °C. A Gigabot 3+ thermoplastic printer from re:3D was used to print the fan mounts for the photoreactor using black PLA (RS stock 832-0264).

Design: Autodesk Fusion 360 was used to design the prints for this project.

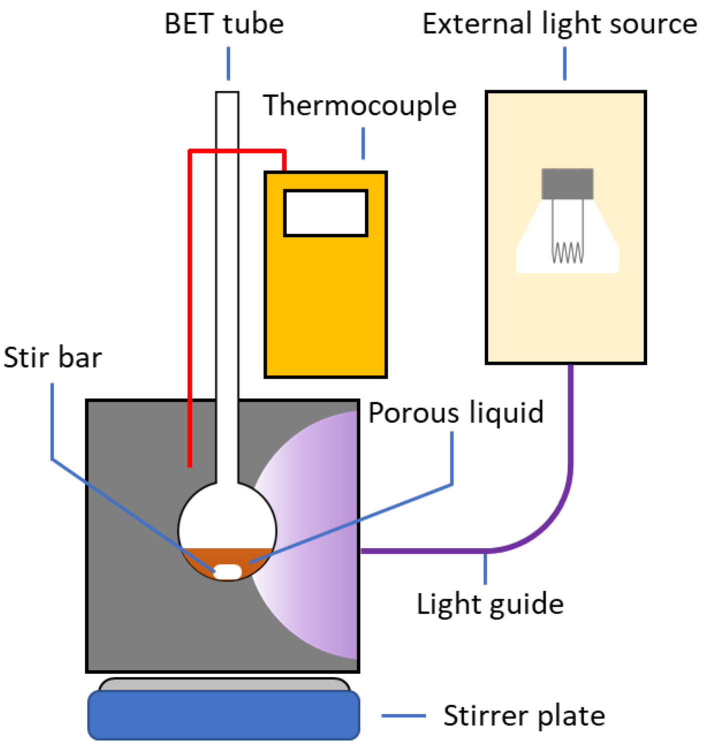

Supplementary Figure 1 Schematic representation of experimental sorption setup.

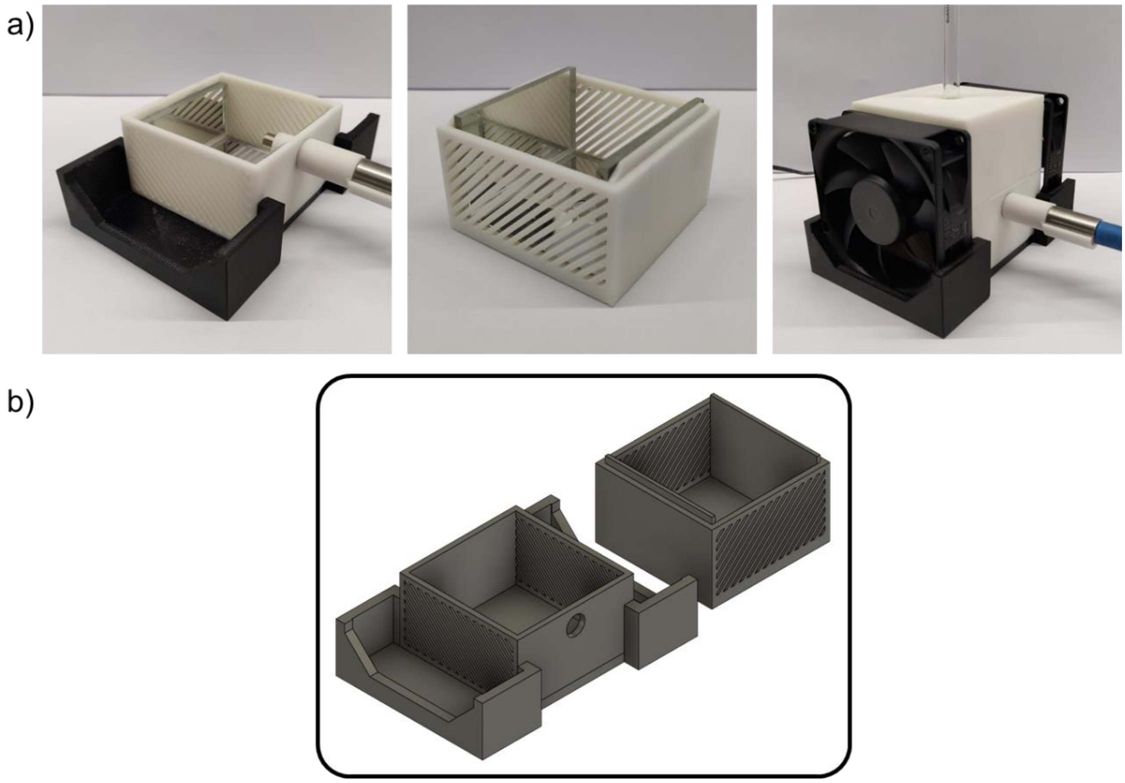

Supplementary Figure 2 a) 3D printed reactor for light-responsive sorption; b) 3D design of light box for sorption setup.

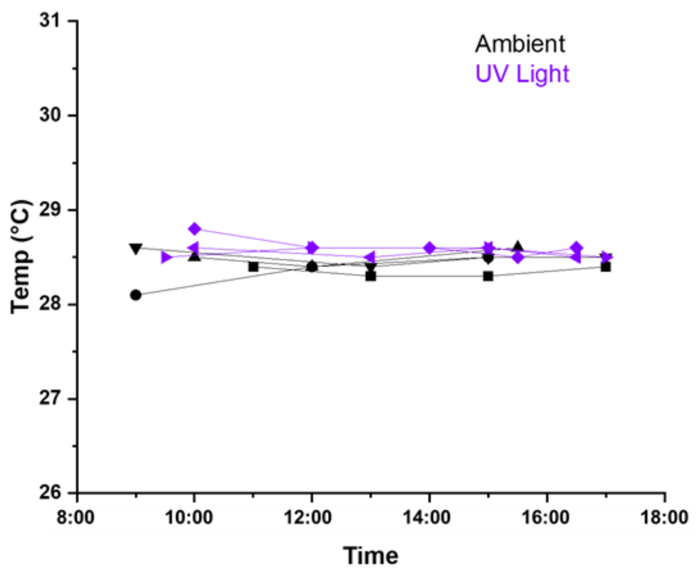

Supplementary Figure 3 Temperature monitoring over time under ambient (absence of light) and UV light within the reactor box.

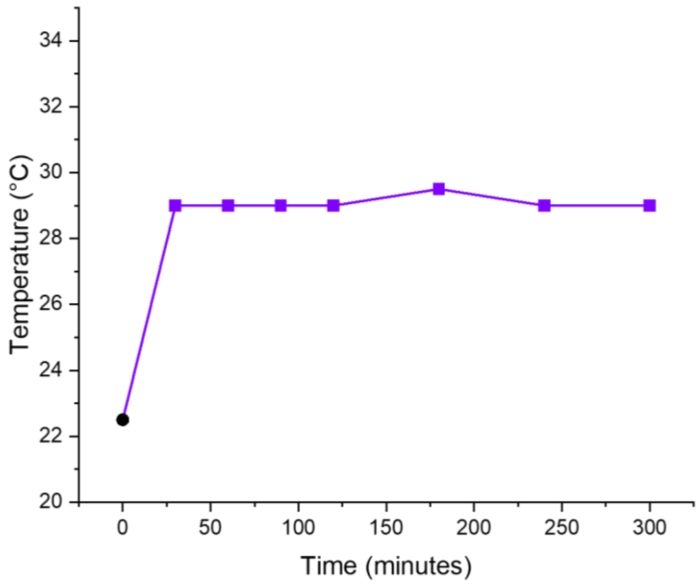

Supplementary Figure 4 Temperature monitoring over time of a porous liquid sample (5 wt. % MOF in [BMIM][NTf_2] under constant irradiation within the sample BET tube and reactor, monitored with a thermometer placed in the liquid.

5. Porous Liquid Solvent Screen

Four solvents were screened that have been previously reported as being size excluded, which include 1-butylpyridinium bis(trifluoromethanesulfonyl)imide ([BPy][NTf$_2$]), 1-ethyl-3-(hydroxymethyl) pyridinium ethyl sulfate ([EtHPy][EtS]), silicone oil AR 20, and 1-butyl-3-methylimidazolium bis(trifluoromethylsulfonyl)imide ([BMIM][NTf$_2$]). All samples were prepared at 12.5 wt. % mixtures and gas uptake measurements were taken under ambient conditions. Of the four samples, both the silicone oil and [BMIM][NTf$_2$] had the highest CO_2 uptake and largest difference between the neat liquid and the 12.5 wt. % sample. These were then investigated for their CH_4 uptake where silicone oil AR 20 was found to have four times the uptake than [BMIM][NTf$_2$]. Therefore, [BMIM][NTf$_2$] was our chosen solvent due to its ability for high CO_2 uptake and minimal CH_4 uptake, allowing us to investigate the possibility of a CO_2/CH_4 selective porous liquid.

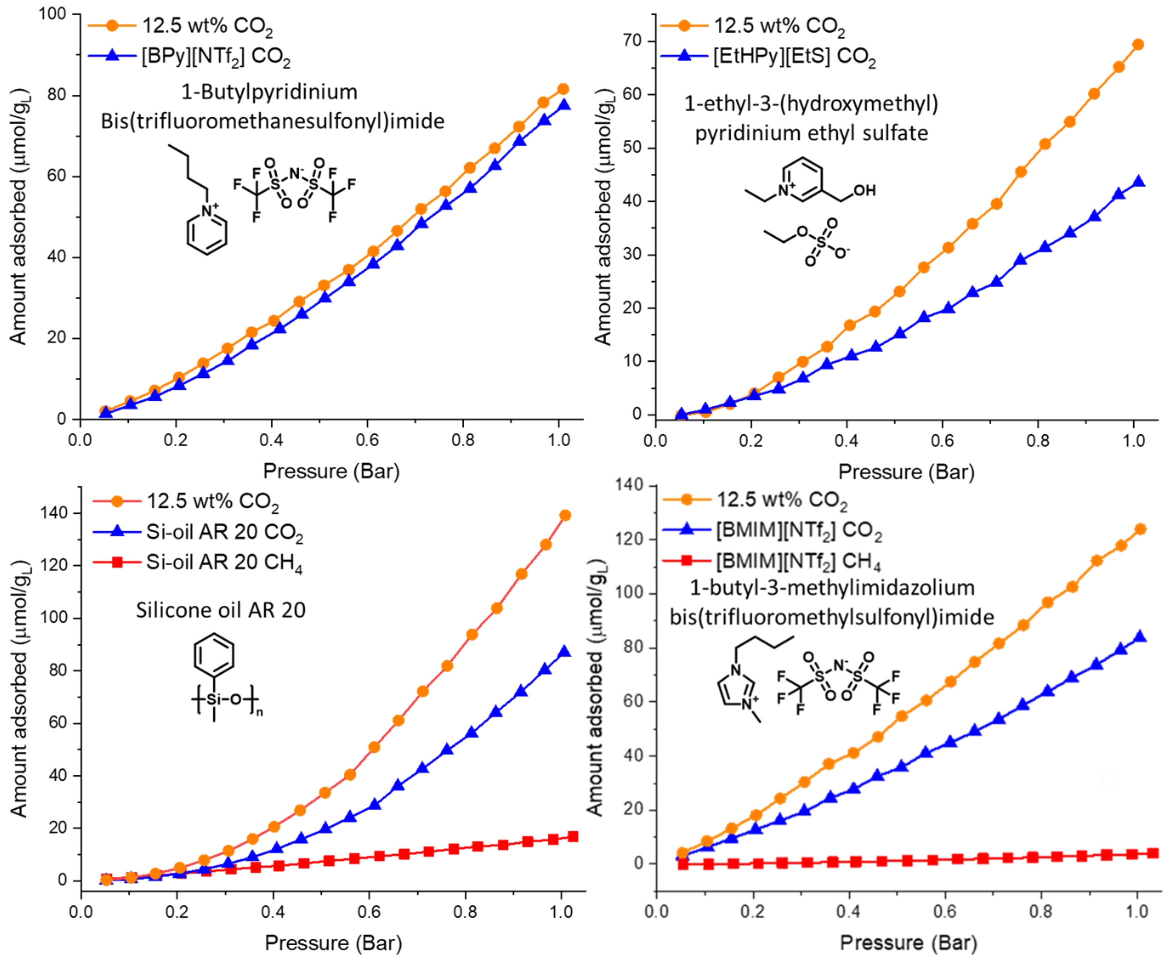

Supplementary Figure 5 Gas uptake measurements to screen for potential porous liquids using 12.5 wt. % Zn(AzDC)(4,4'-BPE)$_{0.5}$ in different ionic liquids and silicone oil AR 20.

6. Material Reproducibility and Gas Sorption Studies

Preliminary experiments were performed with mixed batches of material, which yielded incoherent results. To determine the reproducibility of the bulk material, six batches of MOF were prepared using identical conditions and on the same scale and analysed individually. PXRD analysis showed a variety of different signals, displaying differences across the batches of bulk material. CO_2 sorption measurements (performed on the Quantachrome at RT) also found a broad range of CO_2 uptake in the solid MOF samples, ranging from as little as 277 µmol/g up to 766 µmol/g. Three of these samples were subsequently explored as porous liquids.

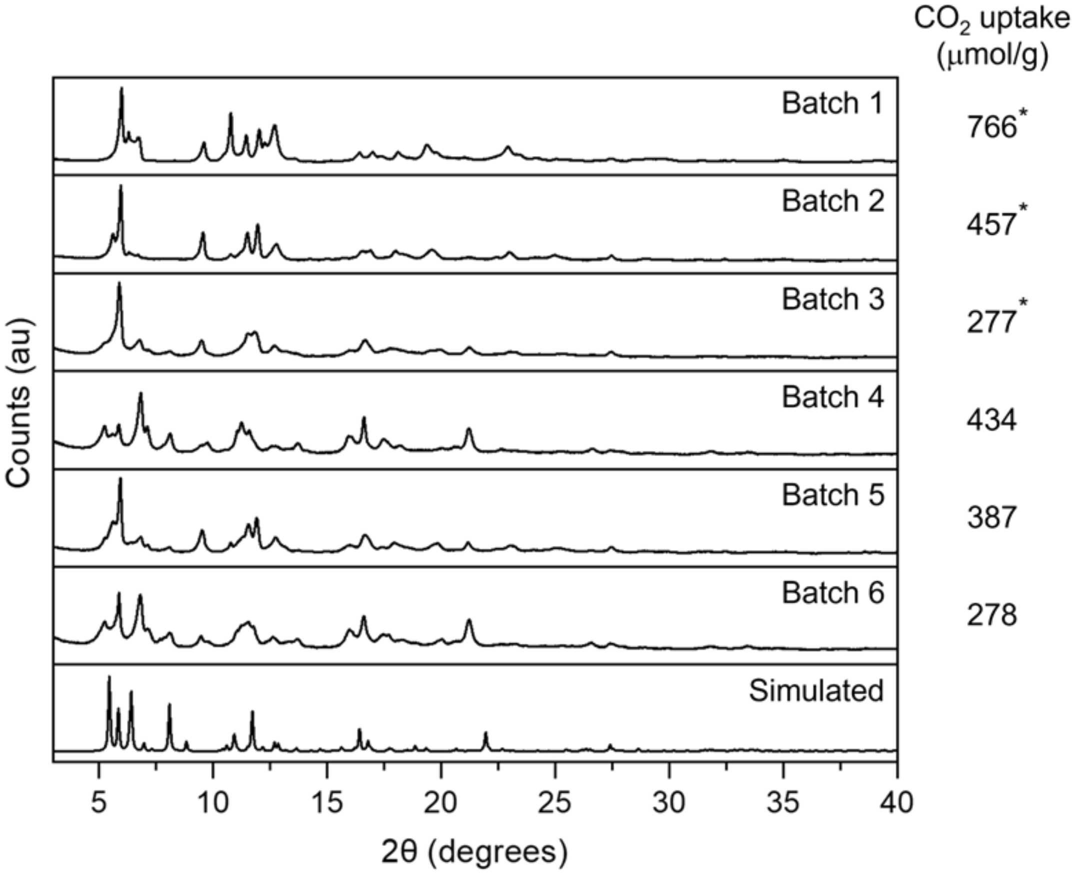

Supplementary Figure 6 PXRD patterns of six individually synthesised batches of Zn(AzDC)(4,4'-BPE)$_{0.5}$ and their corresponding CO_2 uptake in the solid state at 25 °C. Samples marked with an asterisk (*) underwent further testing as porous liquids, noted PL1, PL2, and PL3, from the highest to lowest uptake. Simulated pattern of triply-interpenetrated MOF obtained from scXRD data (CCDC 649367).[2]

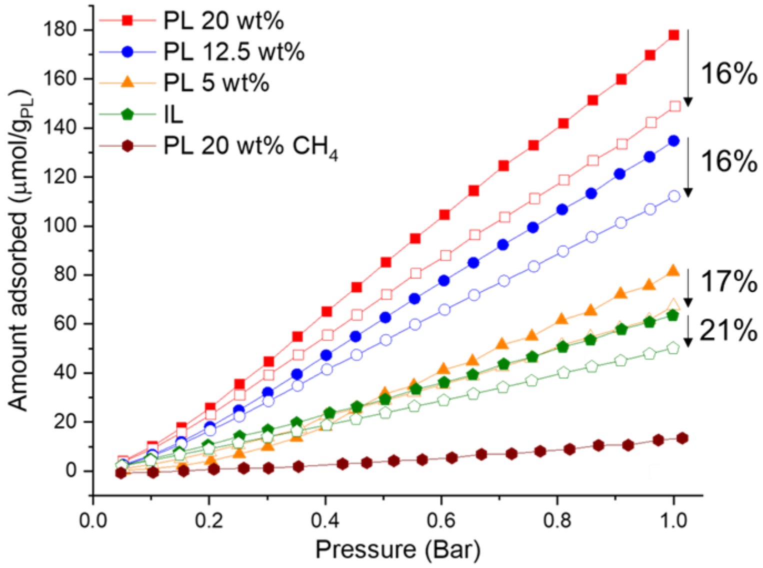

Supplementary Figure 7 CO_2 and CH_4 (labelled) adsorption isotherms of photoresponsive porous liquids prepared at different wt. %'s from batch 1 (PL1), and the neat ionic liquid [BMIM][NTf$_2$], under ambient conditions (filled) and irradiated with UV light (empty).

Supplementary Table 1 Summary of gas uptake measurements of neat ionic liquid, Zn(AzDC)(4,4'-BPE)$_{0.5}$, and the porous liquids produced from batch 1 of the MOF, with their corresponding gas uptakes.

	CO_2 uptake at 1 bar (μmol/g_L)		Predicted uptake at 1 bar (μmol/g_L)		% of predicted uptake at 1 bar	
Sample	Ambient	UV	Ambient	UV	Ambient	UV
PL 20 wt%	178.1	148.8	204.0	145.3	87	102
PL 12.5 wt%	134.8	112.2	151.4	109.6	89	102
PL 5 wt%	81.5	67.4	98. 7	73.9	82	91
MOF	766.7	526.3	-	-	-	-
IL	63.5	50.1	-	-	-	-

Sample	CH_4 uptake at 1 bar (μmol/g_L)	Predicted uptake at 1 bar (μmol/g_L)	% of predicted uptake at 1 bar
PL 20 wt%	13.4	19.4	68
MOF	81.4	-	-
IL	4.0	-	-

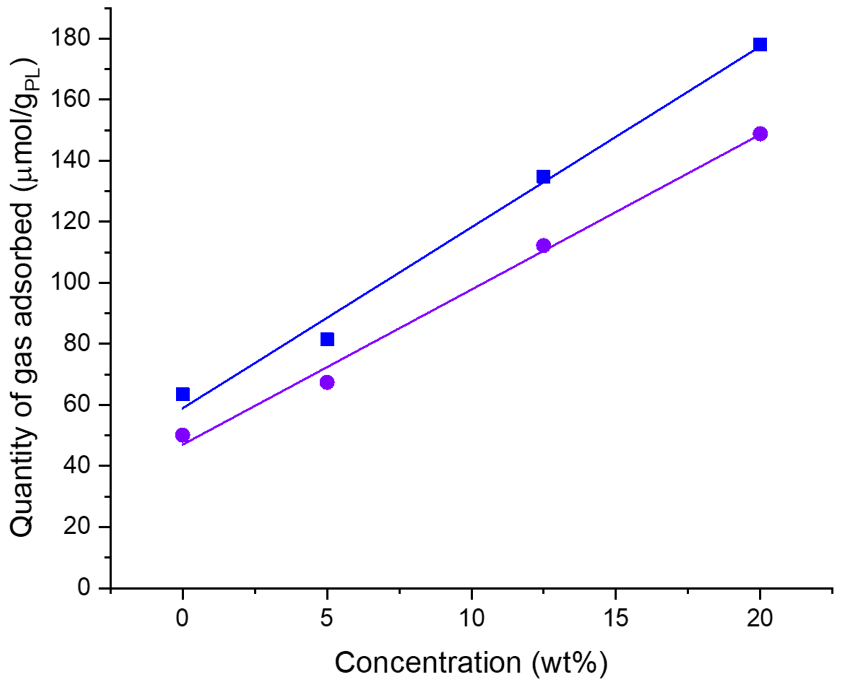

Supplementary Figure 8 Measured gas uptake across a range of concentrations of porous liquid prepared from batch 1 (PL1), under ambient conditions (blue squares) and constant irradiation (purple circles), with the corresponding linear fit lines.

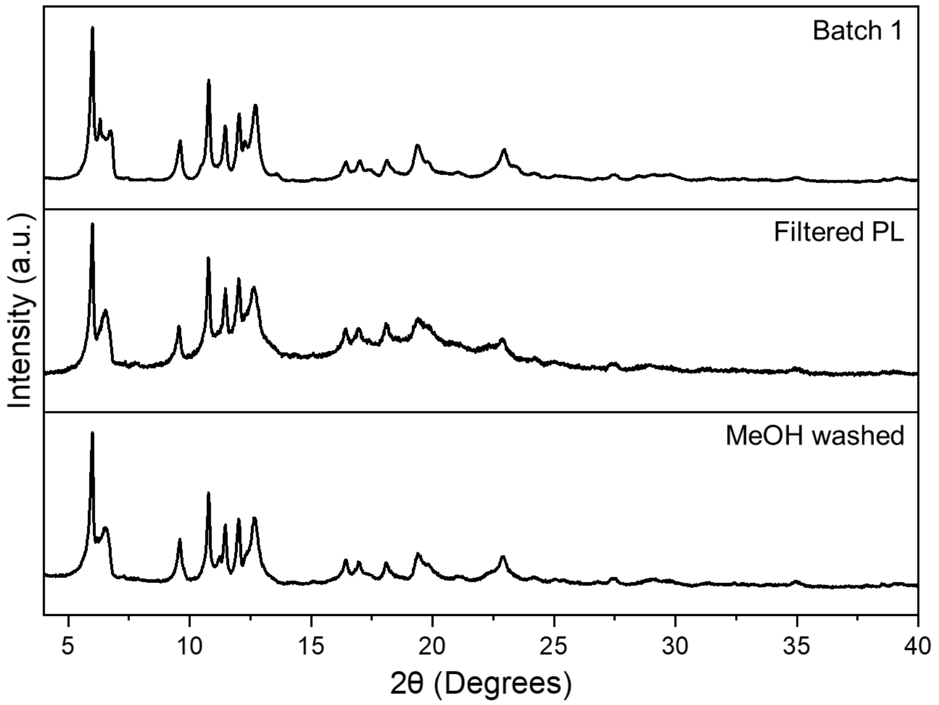

Supplementary Figure 9 PXRD patterns of Zn(AzDC)(4,4'-BPE)$_{0.5}$ (batch 1), the MOF collected by filtration from the porous liquid, and the sample washed with MeOH, after gas sorption experiments.

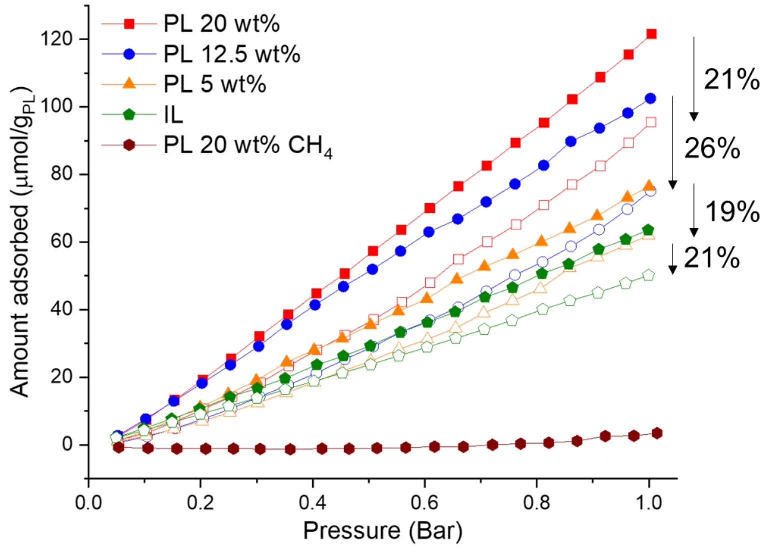

Supplementary Figure 10 CO_2 and CH_4 (labelled) adsorption isotherms of photoresponsive porous liquids prepared at different wt. %'s from batch 2 (PL2), and the neat ionic liquid [BMIM][NTf$_2$], under ambient conditions (filled) and irradiated with UV light (empty).

Supplementary Table 2 Summary of gas uptake measurements of neat ionic liquid, Zn(AzDC)(4,4'-BPE)$_{0.5}$, and the porous liquids produced from batch 2 of the MOF, with their corresponding gas uptakes.

Sample	CO_2 uptake at 1 bar (μmol/g_L)		Predicted uptake at 1 bar (μmol/g_L)		% of predicted uptake at 1 bar	
	Ambient	UV	Ambient	UV	Ambient	UV
PL 20 wt%	121.5	95.4	142.4	108.1	85	88
PL 12.5 wt%	102.5	75.1	112.8	86.3	90	86
PL 5 wt%	76.4	61.9	83.3	64.6	91	95
MOF	457.8	339.8	-	-	-	-
IL	63.5	50.1	-	-	-	-

Sample	CH_4 uptake at 1 bar (μmol/g_L)	Predicted uptake at 1 bar (μmol/g_L)	% of predicted uptake at 1 bar
PL 20 wt%	3.4	12.3	27
MOF	45.8	-	-
IL	4.0	-	-

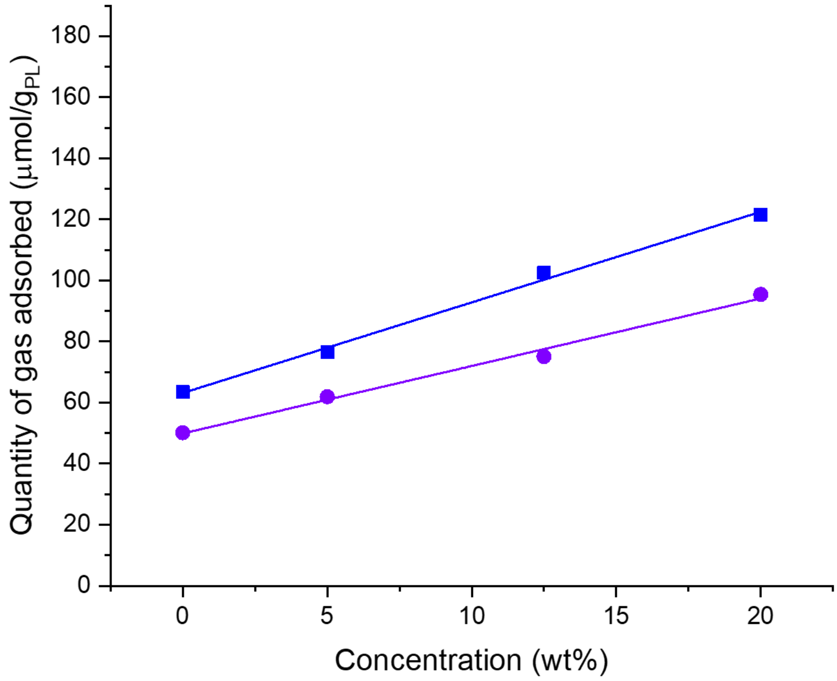

Supplementary Figure 11 Measured gas uptake across a range of concentrations of porous liquid prepared from batch 2 (PL2), under ambient conditions (blue squares) and constant irradiation (purple circles), with the corresponding linear fit lines.

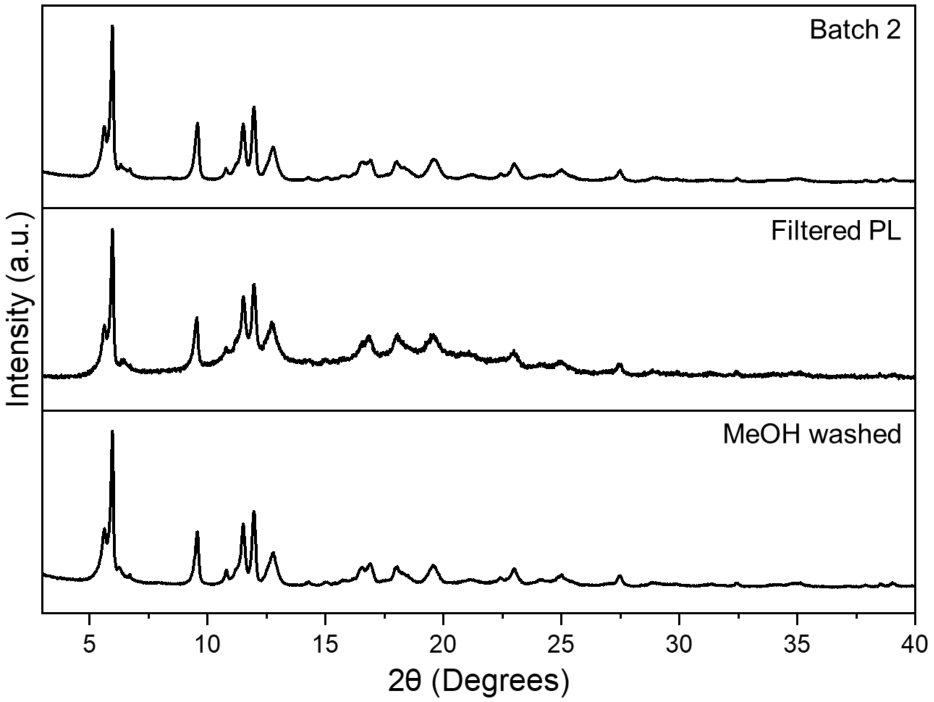

Supplementary Figure 12 PXRD patterns of Zn(AzDC)(4,4'-BPE)$_{0.5}$ (batch 2), the MOF collected by filtration from the porous liquid, and the sample washed with MeOH, after gas sorption experiments.

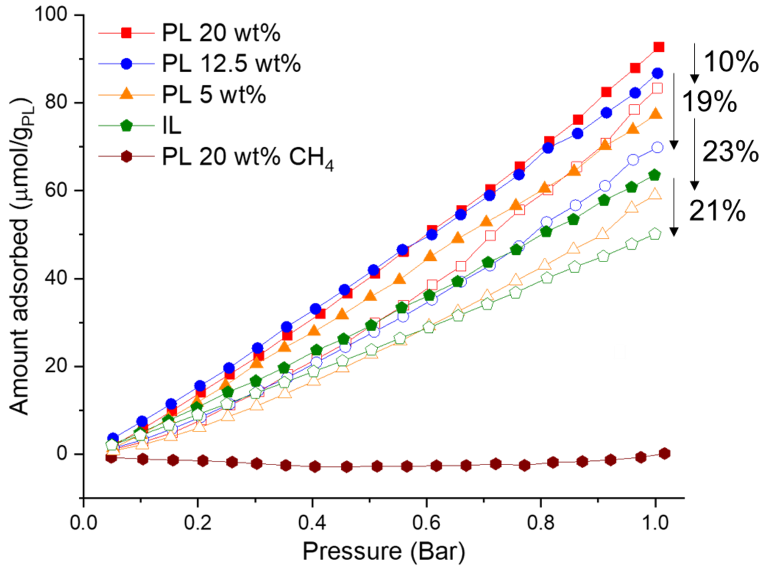

Supplementary Figure 13 CO_2 and CH_4 (labelled) adsorption isotherms of photoresponsive porous liquids prepared at different wt. %'s from batch 3 (PL3), and the neat ionic liquid [BMIM][NTf$_2$], under ambient conditions (filled) and irradiated with UV light (empty).

Supplementary Table 3 Summary of gas uptake measurements of neat ionic liquid, Zn(AzDC)(4,4'-BPE)$_{0.5}$, and the porous liquids produced from batch 3 of the MOF, with their corresponding gas uptakes.

	CO_2 uptake at 1 bar (μmol/g$_L$)		Predicted uptake at 1 bar (μmol/g$_L$)		% of predicted uptake at 1 bar	
Sample	Ambient	UV	Ambient	UV	Ambient	UV
PL 20 wt%	92.7	83.3	106.4	91.3	87	91
PL 12.5 wt%	86.7	69.8	90.3	75.8	96	92
PL 5 wt%	77.2	58.9	74.2	60.4	104	97
MOF	277.9	256.0	-	-	-	-
IL	63.5	50.1	-	-	-	-

Sample	CH_4 uptake at 1 bar (μmol/g$_L$)	Predicted uptake at 1 bar (μmol/g$_L$)	% of predicted uptake at 1 bar
PL 20 wt%	0.14	2.9	4
MOF	14.7	-	-
IL	4.0	-	-

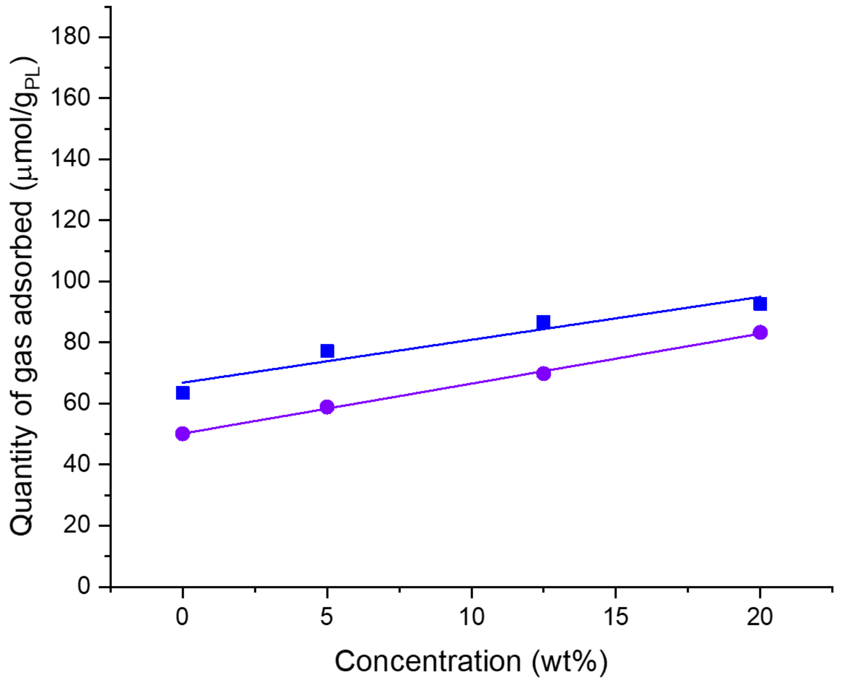

Supplementary Figure 14 Measured gas uptake across a range of concentrations of porous liquid prepared from batch 3 (PL3), under ambient conditions (blue squares) and constant irradiation (purple circles), with the corresponding linear fit lines.

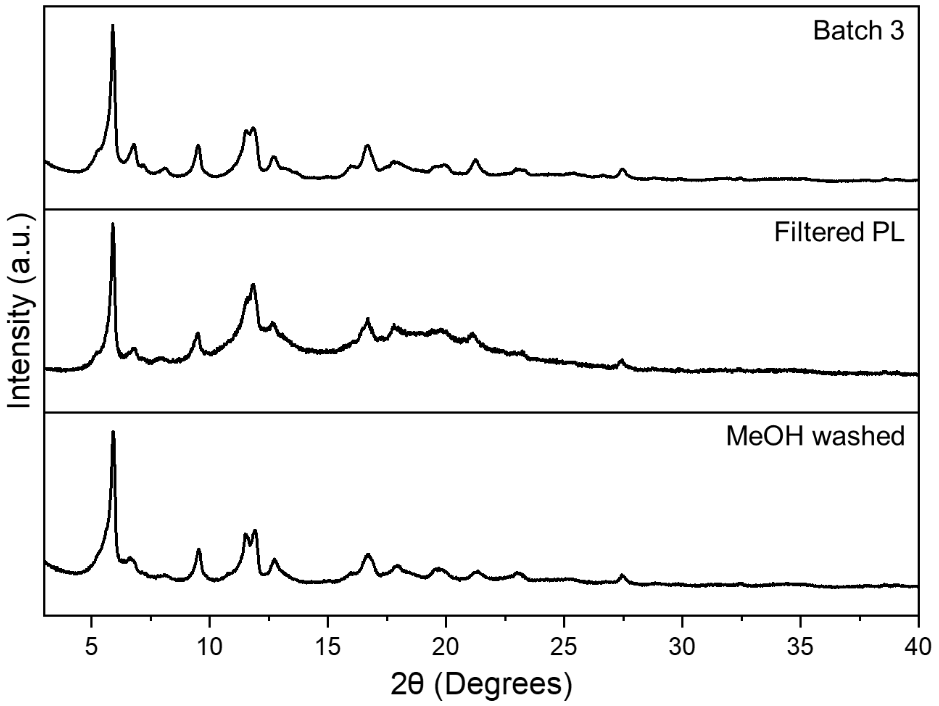

Supplementary Figure 15 PXRD patterns of Zn(AzDC)(4,4'-BPE)$_{0.5}$ (batch 3), the MOF collected by filtration from the porous liquid, and the sample washed with MeOH, after gas sorption experiments.

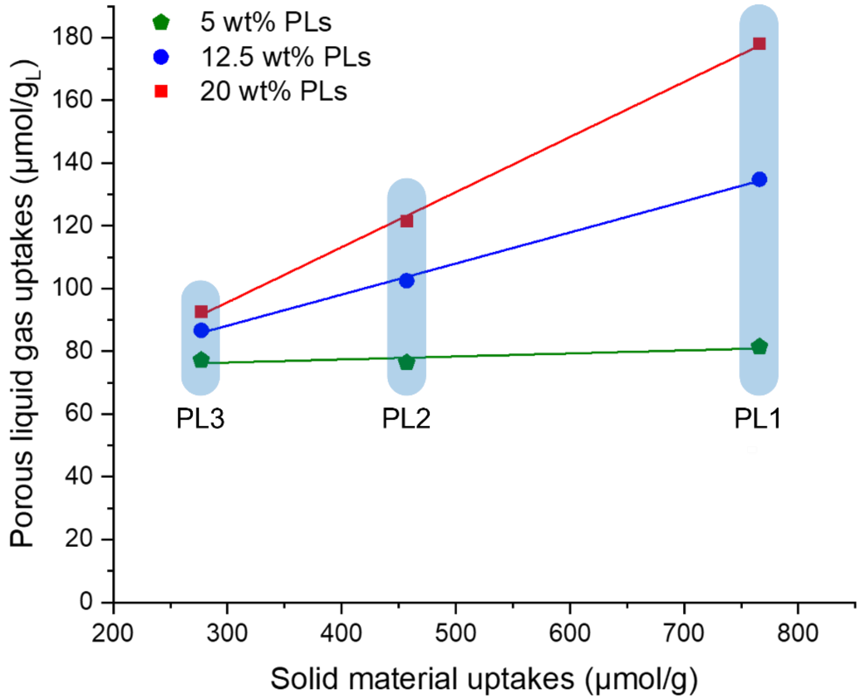

Supplementary Figure 16 Summary of CO_2 uptakes in in batches 1, 2 and 3 of the solid Zn(AzDC)(4,4'-BPE)$_{0.5}$ *vs.* the CO_2 uptake in the corresponding porous liquids at 5 wt. % (green pentagons), 12.5 wt. % (blue circles), and 20 wt. % (red squares).

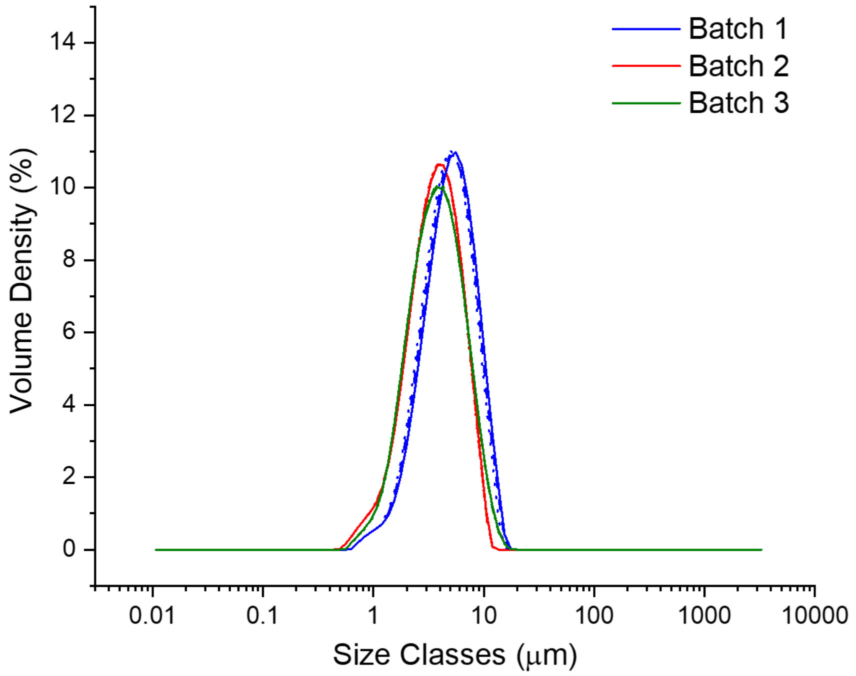

Supplementary Figure 17 SLS of Zn(AzDC)(4,4'-BPE)$_{0.5}$ batches 1, 2, and 3 suspended in MeOH. Each batch was measured 3 times and are plotted as solid, dashed and dotted lines.

Supplementary Table 4 10th, 50th, and 90th percentile values of the particle distribution of Zn(AzDC)(4,4'-BPE)$_{0.5}$ for batches 1, 2, and 3, as measured by SLS in MeOH.

Batch	Dx10	Dx50	Dx90
1	2.31	5.01	9.52
	2.25	4.83	9.23
	2.2	4.68	8.85
2	1.67	3.68	7.05
	1.66	3.65	6.99
	1.66	3.63	6.95
3	1.72	3.73	7.63
	1.72	3.72	7.59
	1.73	3.73	7.6

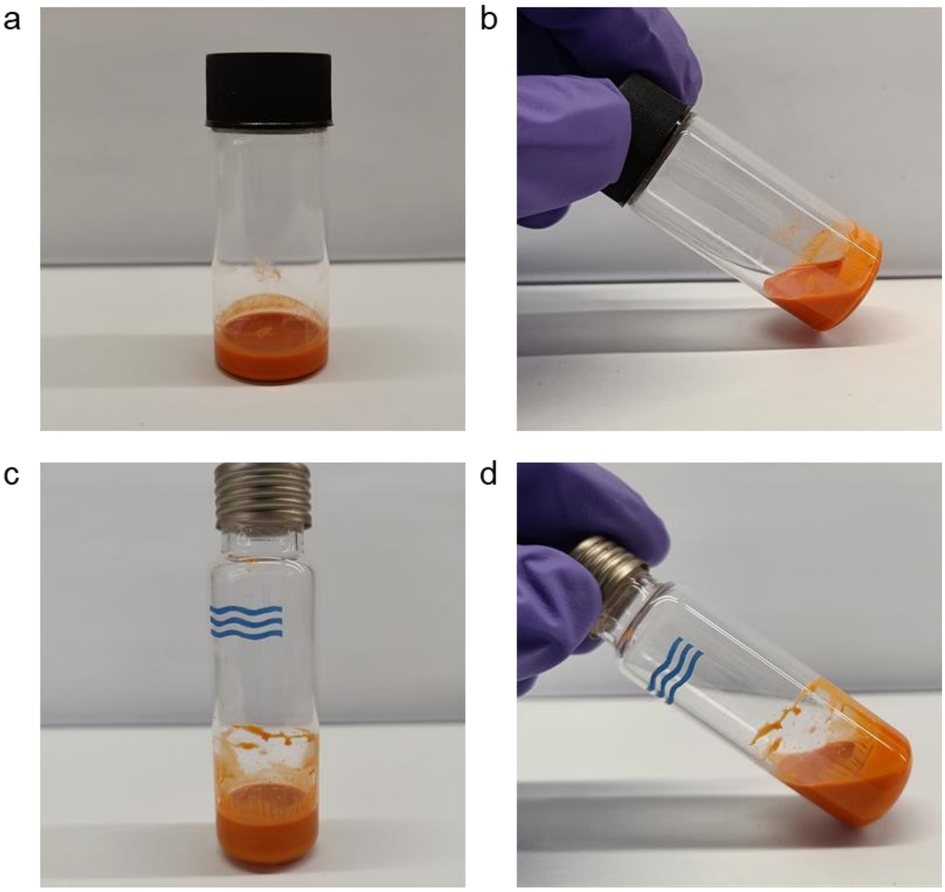

Supplementary Figure 18 Photos showing the stability of porous liquid samples, where: a) 5 wt. % porous liquid after sitting for approximately 6 months; b) as a) with the vial tilted; c) 12.5 wt. % porous liquid after gas displacement measurements with 0.4 mL of $CHCl_3$ in the sample after sitting for approximately 4 months; d) as c) with the vial tilted.

Supplementary Table 5 Viscosity of ionic liquid, [BMIM][NTf_2], and the porous liquid (PL) samples at concentrations of 5, 12.5 and 20 wt. %. Samples were measured at 298 K and a constant shear rate of 300 s^{-1}.

Sample	Viscosity (mPa.s)
[BMIM][NTf_2]	43.9 ± 0.4
5 wt. % PL	74.6 ± 2.3
12.5 wt. % PL	64.5 ± 0.5
20 wt. % PL	244.1 ± 0.9

7. Gas Evolution Experimental setup

Gas Uptake and Evolution Studies: Samples of porous liquids and ionic liquids were tested with gas purchased from BOC with research grade carbon dioxide (N5.0) in GC headspace vials (22 mm x 45 mm screw top, 10 mL, Fisher Scientific). All samples were exposed to a flow of gas bubbled through the liquid and measurements conducted at room temperature (20 – 25 °C) in a temperature-controlled laboratory. Prior to making up the porous liquid sample and carrying out any measurements, both the MOF and ionic liquid were heated to 150 °C in a vacuum oven overnight.

The flow rate of gas was measured using a Gilmont calibrated flowmeter (tube size 0, Gilmont EW-03201-22) with a stainless-steel float on a scale of 1-100. The flow of gas used was in the region that was previously reported for porous liquids and the rate was set to ~50-60 mL/min.[4] Samples were left for 30 minutes under continuous bubbling before measuring gas displacement.

Gas evolution was measured by displacement of water in an inverted burette (0.1 mL graduations) which was placed in a beaker of water connected to a GC vial containing the sample *via* a needle and tubing. Prior to exposing the sample to the selected method, a syringe of air was pushed into the sample vial to remove any residual water in the tube and to set the start point prior to taking the initial measurement. A small amount of vacuum grease was applied to the cap to ensure no holes created by the needle affected the measurement. Measurements were repeated a total of 4 times on the PL1 sample and 3 times on ionic liquid sample for each release method, but only once for chemical displacement measurements on both samples (Supplementary Tables S4-5). Gas evolution measurements were taken after 30 minutes of their corresponding method of release.

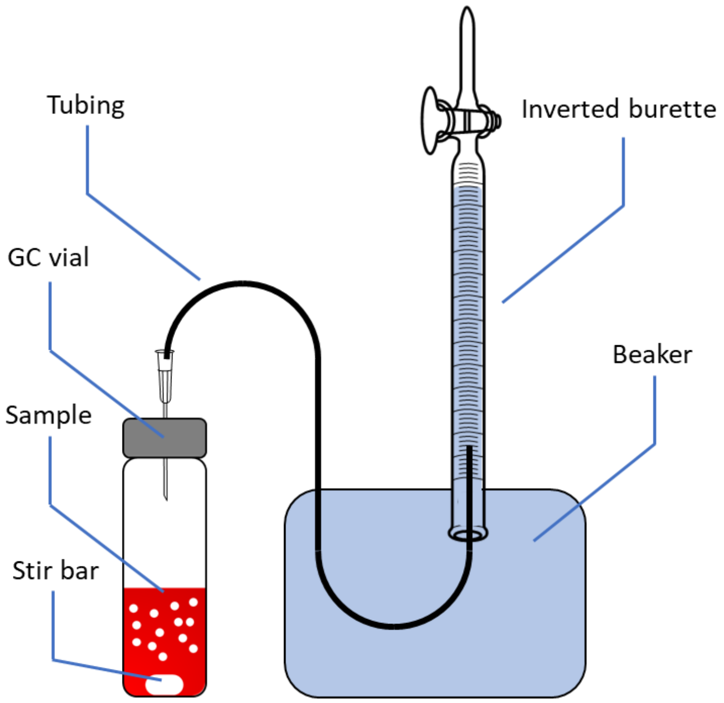

Supplementary Figure 19 Experimental setup for gas evolution.

Supplementary Table 6 Gas evolution data for a 12.5 wt. % porous liquid prepared from batch 1 of the MOF (PL1), at 298 K. Calculations are based on 2.82 g of material.

Method	Volume evolved (mL)	Mean volume evolved (mL)	Gas uptake (μmol/g_{PL})
UV	4.95	5.05 ± 0.55	79.8 ± 8.78
	5.20		
	4.25		
	5.80		
Sonication	5.90	5.20 ± 0.46	82.2 ± 7.33
	5.10		
	4.60		
	5.20		
Heat	6.40	6.23 ± 0.24	98.6 ± 3.92
	6.50		
	5.85		
	6.20		
Chemical displacement	4.80	4.80	75.9

Supplementary Table 7 Gas evolution data for the ionic liquid [BMIM][NTf_2]. Calculations are based on 5.11 g of material.

Method	Volume evolved (mL)	Mean volume evolved (mL)	Gas uptake (μmol/g_{PL})
UV	1.90	1.73 ± 0.23	15.1 ± 2.05
	1.90		
	1.40		
Sonication	2.80	3.26 ± 1.11	28.5 ± 9.69
	2.20		
	4.80		
Heat	4.70	4.43 ± 0.25	38.7 ± 2.17
	4.10		
	4.50		
Chemical displacement	2.40	2.40	17.4

Supplementary Table 8 Comparison of gas evolution experiments performed on a 12.5 wt. % porous liquid sample (PL1) and the neat ionic liquid, [BMIM][NTf_2], with the working capacity (the difference in uptake of the porous liquid (PL) and ionic liquid (IL)) and the percentage difference in release of CO_2.

Gas release mechanism	Gas released (μmol/g)		Working capacity, PL-IL (μmol/g)	% Difference in release
	PL	IL		
UV	79.84	15.11	64.73	81
Sonication	82.21	28.48	53.73	65
Heat	98.62	38.66	59.96	61
Displacement	75.9	17.44	58.46	77

8. UV-Vis and NMR Study of [BMIM][NTf_2]

To explore [BMIM][NTf_2] using UV-Vis spectroscopy (Supplementary Figure 17), the material was dissolved in acetonitrile – the absorbance spectra showed very similar features to [BMIM][BF_4],[5] with no absorbance in the visible wavelength, and an increase in absorbance as the wavelength falls below 400 nm, which likely corresponds to the $\pi \rightarrow \pi^*$ transition, where acetonitrile is featureless until below 250 nm (note – the small change at 350 nm is due to the light source changing to the lower range wavelength during the experiment).

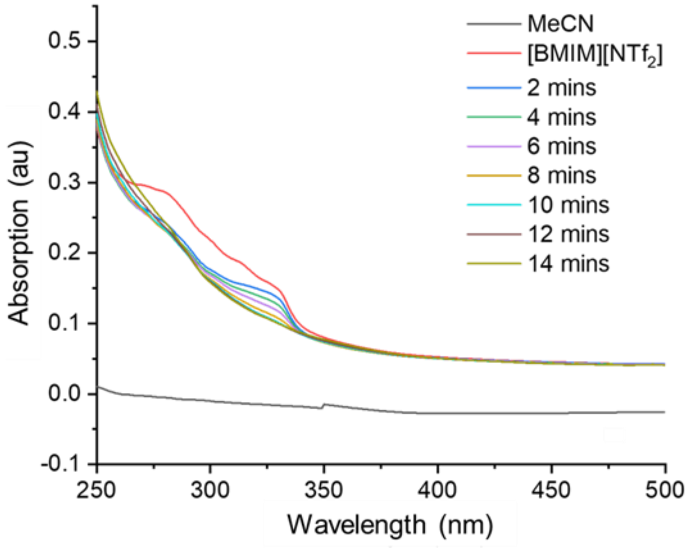

Supplementary Figure 20 Absorption spectra of acetonitrile (grey) and [BMIM][NTf_2] in acetonitrile, before (red) and after irradiation with 2-minute intervals with cumulative irradiation times shown in minutes.

Rather than irradiating the sample at the λ_{max}, the sample was irradiated with the same light source (365 nm) as the photoswitching porous liquid experiments to determine whether there were any changes under similar conditions as the gas sorption experiments. The sample was irradiated in 2-minute intervals, up to 14 minutes. After irradiating the sample for 2 minutes, there was a significant change in the absorbance where the λ_{max} at 280 nm decreased along with a decrease in absorbance between 260 nm – 330 nm. After continued irradiation, this trend continues up to 10 minutes, although there is a much slower change at 330 nm, whereas the initial λ_{max} at 280 nm changes very little over the course of continued irradiation. After 10 minutes there were no further appreciable changes in the spectra.

A ^1H NMR spectroscopic study was also performed in acetonitrile, where the same sample was analysed under ambient conditions and after irradiation for 10 minutes. In both cases no change was observed in the ^1H NMR spectra, including the sample concentration which

remained constant and determined by integrating the aromatic protons and the residual solvent signal. Although there is no conclusive evidence of mass structural change, there is evidence of a change taking place in the material as observed in the UV-Vis experiments, which may explain the difference in the observed CO_2 uptake while the sample is being irradiated.

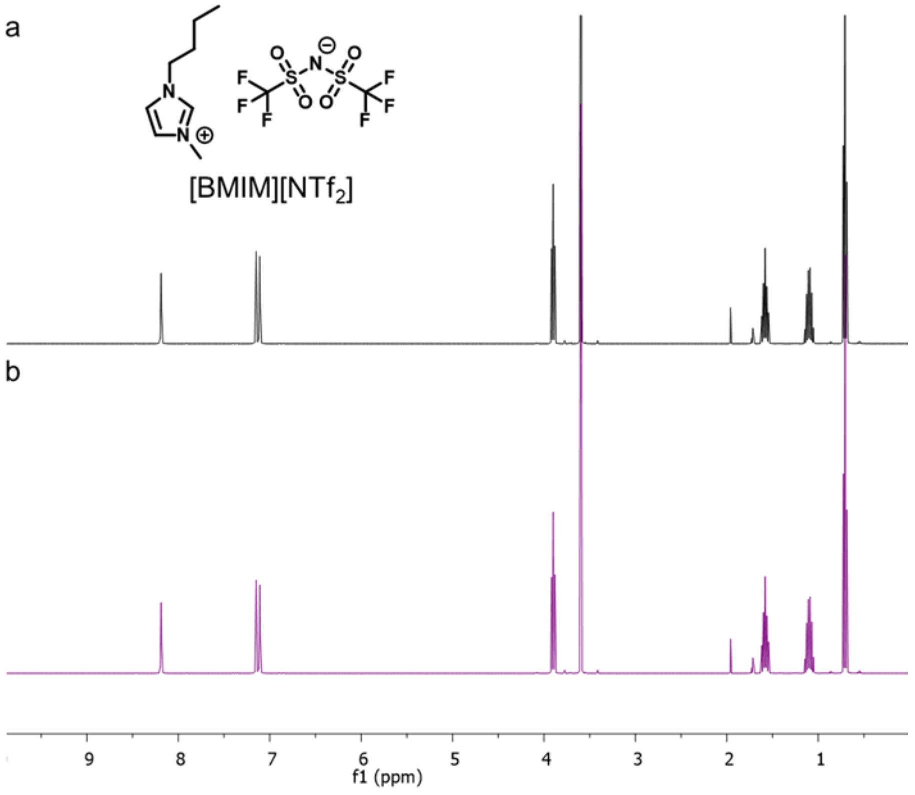

Supplementary Figure 21 ^{1}H NMR spectra of [BMIM][NTf$_2$] in acetonitrile: a) performed under ambient conditions; b) sample irradiated for 10 minutes at 365 nm.

9. References

1 R. Lyndon, K. Konstas, B. P. Ladewig, P. D. Southon, P. C. J. Kepert and M. R. Hill, *Angew. Chemie Int. Ed.*, 2013, **52**, 3695–3698.

2 B. Chen, S. Ma, E. J. Hurtado, E. B. Lobkovsky and H. C. Zhou, *Inorg. Chem.*, 2007, **46**, 8490–8492.

3 A. Kai, B. D. Egleston, A. Tarzia, R. Clowes, M. E. Briggs, K. E. Jelfs, A. I. Cooper, R. L. Greenaway, A. Kai, B. D. Egleston, R. Clowes, M. E. Briggs, A. I. Cooper, R. L. Greenaway, A. Tarzia and K. E. Jelfs, *Adv. Funct. Mater.*, 2021, **31**, 2106116.

4 R. L. Greenaway, D. Holden, E. G. B. Eden, A. Stephenson, C. W. Yong, M. J. Bennison, T. Hasell, M. E. Briggs, S. L. James and A. I. Cooper, *Chem. Sci.*, 2017, **8**, 2640–2651.

5 L. Rao, W. Wei, Z. Xia, F. Li, F. Yang and K. Zhou, *Asian J. Phys. Chem. Sci.*, 2017, **4**, 1–12.
